# Altered Neurocritical Care Management of Patients with Severe Traumatic Brain Injury Following Changed Positions of the Zero-Reference Points for Intracranial and Arterial Pressure Measurement

**DOI:** 10.1007/s12028-025-02366-2

**Published:** 2025-09-08

**Authors:** Linus Réen, Hannes Wikström, Edward Visse, David Cederberg, Peter Siesjö, Niklas Marklund

**Affiliations:** 1 Department of Clinical Sciences Lund, Neurosurgery, Department of Clinical Sciences, Lund University, Lund, Sweden; 2 Department of Neurosurgery, Skåne University Hospital, Lund, Sweden

**Keywords:** Traumatic brain injury, Intracranial pressure, Cerebral perfusion pressure, Neurocritical care, Reference point

## Abstract

**Background:**

Many traumatic brain injury (TBI) treatment protocols, including the Lund concept, advocate the highest point of the subarachnoid space (typically the vertex) as the zero-reference point for intracranial pressure (ICP) and the level of the right atrium as the zero-reference point for mean arterial blood pressure (MAP). In 2017, at the Department of Neurosurgery in Lund, Sweden, the zero-reference points for ICP and MAP were both changed to the external auditory meatus (EAM), thus altering the calculated cerebral perfusion pressure (CPP) levels. We hypothesized that the ICP and MAP levels obtained from the different zero-reference points resulted in altered neurocritical care management and/or patient outcome.

**Methods:**

We conducted a retrospective analysis of ICP, CPP, MAP, medical management, mortality, and outcome in two different patient cohorts with severe TBI treated at the Department of Neurosurgery, Skåne University Hospital, Lund, Sweden, between 2013 and 2016 and 2018 and 2022.

**Results:**

We collected more than 31,000 measurements from 49 patients between 2013 and 2016 and 53 patients between 2018 and 2022. Age and injury severity were similar in both groups. Mortality and treatment outcome according to the Glasgow Outcome Scale – Extended were similar. Mean ICP levels were higher (*p* < 0.0001) after the reference point was changed to the EAM. The use of clonidine (65% vs. 49%; *p* = 0.17) and metoprolol (50% vs. 13%; *p* = 0.0002) decreased, and the use of norepinephrine increased (42% vs. 98%; *p* < 0.0001) after changing the reference points.

**Conclusions:**

Higher ICP levels were observed when the reference point was changed to the EAM. The use of metoprolol was reduced, and there was a significant increase in the use of norepinephrine. These results show the impact of zero-reference point placement, which should be reported in TBI studies analyzing ICP and CPP management.

## Introduction

Since the first ever direct measurements of intracranial pressure (ICP) in the 1950s by Guillaume and Janny [1] and the development of continuous ICP recording in the 1960s by Lundberg [2], the perception of ICP monitoring has evolved from a highly invasive and feared procedure to an essential component of modern neurocritical care (NCC) for the management of traumatic brain injury (TBI) [3–6]. ICP is most commonly measured using an intraparenchymal microsensor in the frontal lobe or by an intraventricular catheter placed in the frontal horn of the lateral ventricle and connected via a fluid-filled system to an external pressure gauge [7]. Although considerable variation in clinical practice exists, the latter method is frequently preferred, as it not only measures ICP but also allows for in vivo calibration and therapeutic drainage of cerebrospinal fluid [7–9]. Normal values for ICP are commonly reported between 7 and 15 mm Hg [10, 11]. According to available guidelines, treatment is recommended for ICP values > 22 mm Hg or for sustained ICP elevations > 20–25 mm Hg lasting more than five minutes [3, 12].

The cerebral perfusion pressure (CPP) is calculated by subtracting ICP from the mean arterial pressure (MAP). Continuous monitoring of CPP is widely recommended in patients with severe TBI as a surrogate measure of cerebral blood flow [3, 13–17]. However, there is a lack of consensus regarding the optimal CPP thresholds, and current literature recommendations supporting a CPP of 60–70 mm Hg are not substantiated by high-quality evidence [3, 14, 18–20]. Additionally, clinical guidelines for CPP are derived from studies that either employ different reference points for MAP or fail to specify the level of the reference point [21, 22].

Several anatomical landmarks have been suggested as reference points for ICP and MAP. Although there is currently no consensus, the most frequently discussed in the literature are the vertex or the foramen of Monro for ICP and the phlebostatic axis (PA)—i.e., the level of the right atrium—for MAP and, consequently, CPP [5, 13, 15, 21, 23]. Previous studies have demonstrated significant variability in the reference points used for ICP and MAP measurement, with variations observed also within the same hospital [13, 21, 24]. The use of PA as a reference point for MAP has been criticized in the management of TBI, as it can theoretically overestimate CPP up to 18 mm Hg, thereby risking secondary ischemia [15, 21, 23]. In addition to the positioning of the reference point, the degree of head elevation needs to be considered when ICP is monitored [25]. A common practice in the neurointensive care unit (NICU) is a head elevation of 30 degrees [26], as it has shown to decrease ICP without significantly affecting CPP [25]. This effect is, in part, achieved because of increased venous outflow; however, the exact mechanisms are not fully understood [27], and previous studies have shown a risk of low CPP and a paradoxical increase in ICP [28, 29] with head elevation in patients with severe TBI.

**Fig. 1:**
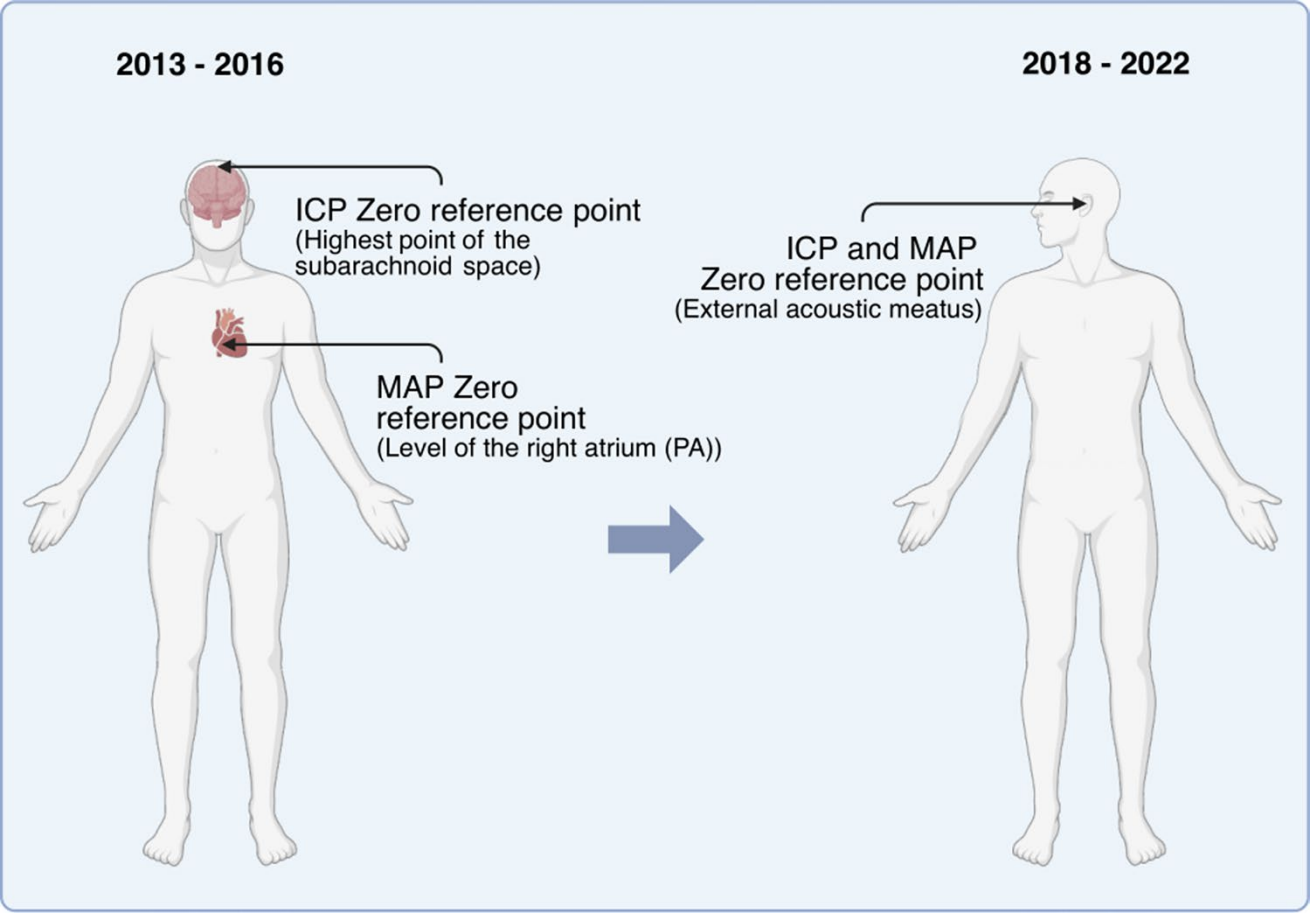
Illustration of the different reference points used in patients treated between 2013 and 2016 and 2018 and 2022. Created with BioRender.com. ICP intracranial pressure, MAP mean arterial pressure, PA phlebostatic axis

Patients with TBI admitted to the NCC unit at Skåne University Hospital have since long been managed according to the Lund concept [30, 31], using the highest point of the subarachnoid space (typically the vertex) as the reference point for ICP and the PA as the reference point for MAP. The Lund concept treatment protocol has been debated [32, 33], potentially because it may underestimate ICP due to the positioning of the ICP transducer at the vertex and overestimate CPP due to the positioning of the MAP transducer at the PA. In 2017, the treatment protocol for severe TBI at the Department of Neurosurgery in Lund, Sweden, was revised by the chief medical physician of the NICU following a careful evaluation of the available literature. As part of this update, the reference points for ICP and MAP were changed to the external acoustic meatus (EAM) (Fig. 1). The aim of this study was to compare ICP and CPP from the different reference points and to investigate how treatment and outcome were affected by the change of reference points.

## Methods

This retrospective observational study included patients with severe TBI who were treated at the Department of Neurosurgery, Skåne University Hospital, between 2013 and 2016 and 2018 and 2022. Patients treated between 2013 and 2016 had the zero-reference points for ICP and MAP at the vertex and at heart level, respectively, and patients treated between 2018 and 2022 had the EAM as the zero-reference point for ICP and MAP. Inclusion criteria were patients with severe TBI, defined as a GCS score < 9 within 24 h, requiring ICP monitoring for more than 24 h. Patients with an initial GCS > 9 who rapidly deteriorated and showed clinical and/or radiological signs of raised ICP and were subjected to early interventions (e.g., intubation, hyperventilation, osmotherapy) were also included. Patient characteristics (age, sex, medical history, GCS, and surgical treatment) were collected from medical records (Melior, Cerner). Radiological diagnosis and Marshall classification [34] were collected from the hospital’s radiology software (Sectra IDS7). Physiological variables (e.g., ICP, CPP, and MAP) and total doses of barbiturates, metoprolol, clonidine, and norepinephrine were obtained from IntelliSpace Critical Care & Anesthesia (Philips). Total monitoring hours of ICP, CPP, and MAP were noted, and physiological values were recorded hourly for a maximum of 7 days. Intraparenchymal ICP measurements and values obtained via open ventricular drains were excluded from ICP calculations because intraparenchymal pressures are unaffected by changes in the reference level of the intraventricular transducer, and measurements obtained from open ventricular drains may lack accuracy [35]. We included CPP values derived from both intraventricular and intraparenchymal ICP measurements, as we expected a change in CPP regardless of whether ICP was obtained from intraventricular or intraparenchymal monitoring, given that the MAP reference point was also changed. Patient outcome according to the Glasgow Outcome Scale – Extended (GOSE) [36] and 30-day mortality were obtained from medical records. The median follow-up time was 6 (2–10) years post injury. Physiological parameters and patient characteristics were compared between patients before (2013–2016) and after (2018–22) adjustments to the reference points for ICP and MAP were made. We allowed a one-year run-in phase; therefore, data from 2017 were not included in the analysis. The rationale for comparing the four years before the adjustments with the five years after was to achieve comparable cohort sizes, given the gradual decline in the number of patients with severe TBI treated in our department.

### Statistical Analysis

The Shapiro–Wilk test was used to test data for normality. Nonnormally distributed data were expressed by medians with IQRs. The Wilcoxon signed-rank test was used for nonnormally distributed continuous variables. The χ^2^ test was used for categorical data. For normally distributed data, Welch’s *t*-test was used. Normally distributed data were expressed by means and standard deviations. All computations were run with the free statistical software R-project (https://www.r-project.org).

### Statistical Disclosure Control

All data were deidentified and are presented only at the group level. Data may be shared upon reasonable request; however individual patients cannot be identified. All data were reviewed to ensure compliance with GDPR and institutional data governance policies.

### Ethics

Local permission for this study was provided by *Kvalitetsregister, vårdinformationssystem och beredning* (KVB), Region Skåne. Informed consent was not applicable. The Swedish Ethical Review Authority has approved that NICU data from all patients treated at the Department of Neurosurgery in Lund, Sweden, may be used in retrospective analyses (reference number: 2017/469).

### Artificial Intelligence

The grammar and spelling of this study have, in part, been reviewed using ChatGPT (https://openai.com/index/chatgpt/ChatGPT | OpenAI). The authors have processed the generated text and images and take full responsibility for the content.

## Results

### Patient Characteristics

We identified 49 patients with severe TBI between 2013 and 2016 and 53 patients between 2018 and 2022 who met the inclusion criteria. There was a high male predominance in patients treated between 2013 and 2016 (80%) and in patients treated between 2018 and 2022 (66%; Table 1). Neither age, GCS, Marshall classification, nor the use of barbiturates or the rate of decompressive craniectomy differed significantly between the two groups. Patients treated between 2013 and 2016 had a statistically significantly longer NCC unit stay, resulting in longer duration with MAP monitoring. However, there was no significant difference in ICP or CPP monitoring times. The two cohorts had a similar distribution of intracranial pathologies and radiological classification, and there was no significant difference in outcome or 30-day mortality. GOSE could not be obtained in five (10%) patients treated between 2013 and 2016 and in six (11%) patients treated between 2018 and 2022. One patient treated between 2013 and 2016 was not included in the mortality analysis due to missing data (Table 1).

**Table 1:** Patient characteristics in the two cohorts (2013–2016 and 2018–2022)

	2013–2016	2018–2022	*p* value
Age, median (IQR), years	43 (26–63)	55 (40–64)	0.17
Male, *n*	39	35	0.19
Female, *n*	10	18	0.19
Marshall 1, *n*	0	1	0.999
Marshall 2, *n*	25	19	0.18
Marshall 3, *n*	3	1	0.55
Marshall 4, *n*	0	1	0.999
Marshall 5, *n*	21	30	0.23
Marshall 6, *n*	0	1	0.999
GCS, median (IQR)	8 (8–14)	7 (5–14)	0.6
EDH, *n*	5	6	0.999
SDH, *n*	35	38	0.999
TSAH, *n*	39	34	0.13
Contusion/tICH, *n*	44	38	0.9
DC, *n*	2	1	0.98
Intraventricular ICP monitoring, *n*	28	28	0.8
Median length of stay (IQR), hours	264 (192–471)	192 (96–336)	**0.02**
ICP monitoring, median (IQR), hours	106 (75–201)	171 (68–248)	0.43
CPP monitoring, median (IQR), hours	189 (89–243)	113 (55–254)	0.1
MAP monitoring, median (IQR), hours	222 (130–360)	116 (59–255)	**0.005**
30-day mortality, %	19	25	0.6
Favorable outcome (GOSE 5–8), %	50	47	0.9
Unfavorable outcome (GOSE 1–4), %	50	53	0.9

The Wilcoxon signed-rank test was used for continuous variables, and the χ^2^ test was used for categorical data

CPP, cerebral perfusion pressure, DC, decompressive hemicraniectomy, EDH, epidural hematoma, GCS, Glasgow Coma Scale, GOSE, Glasgow Outcome Scale – Extended, ICP, Intracranial pressure, IQR, Interquartile range, MAP, mean arterial pressure, SDH, subdural hematoma, tICH, traumatic intracerebral hemorrhage, TSAH, traumatic subarachnoid hemorrhage

### ICP Levels

**Fig. 2:**
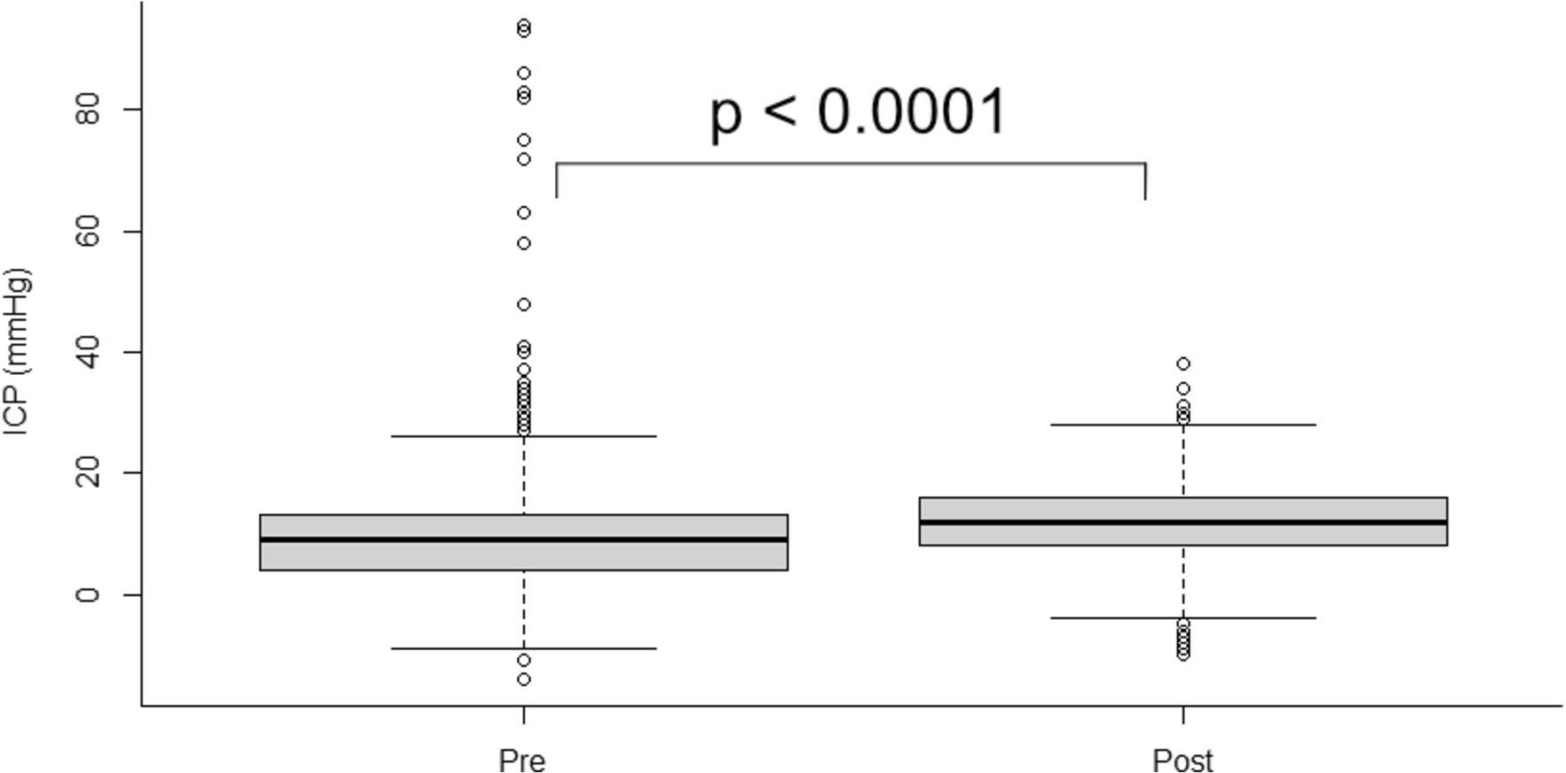
ICP values from intraventricular catheters with closed drainage in patients treated between 2013 and 2016 (pre, 3,369 values) and 2018 and 2022 (post, 3,025 values). ICP was collected hourly for a maximum of 7 days. Median ICP was significantly lower in patients treated between 2013 and 2016 (median 9 vs. 12 mm Hg). Data are shown as median, interquartile range, and range. ICP = intracranial pressure

ICP values were collected from intraventricular catheters with closed drainage from 28 patients between 2013 and 2016 and 28 patients between 2018 and 2022. As previously stated, patients treated between 2013 and 2016 did not differ significantly in total ICP monitoring times compared to those treated between 2018 and 2022 (median 106 h [IQR 75–201 h] and 171 h [IQR 68–248 h], respectively; *p* = 0.4). During the first 7 days, monitoring durations ranged from 24 to 168 h and 25 to 168 h in the two cohorts, respectively. Total monitoring times amounted to 3,482 and 3,476 h in the two cohorts, respectively. In total, 3,369 ICP values were collected in the earlier cohort and 3,025 ICP values were collected in the later cohort. Median ICP was significantly higher (12 mm Hg [IQR 8–16 mm Hg] vs. 9 mmHg [IQR 4–13 mm Hg]; *p* < 0.0001) in patients with EAM as the reference point for ICP (Fig. 2).

### CPP Levels

**Fig. 3:**
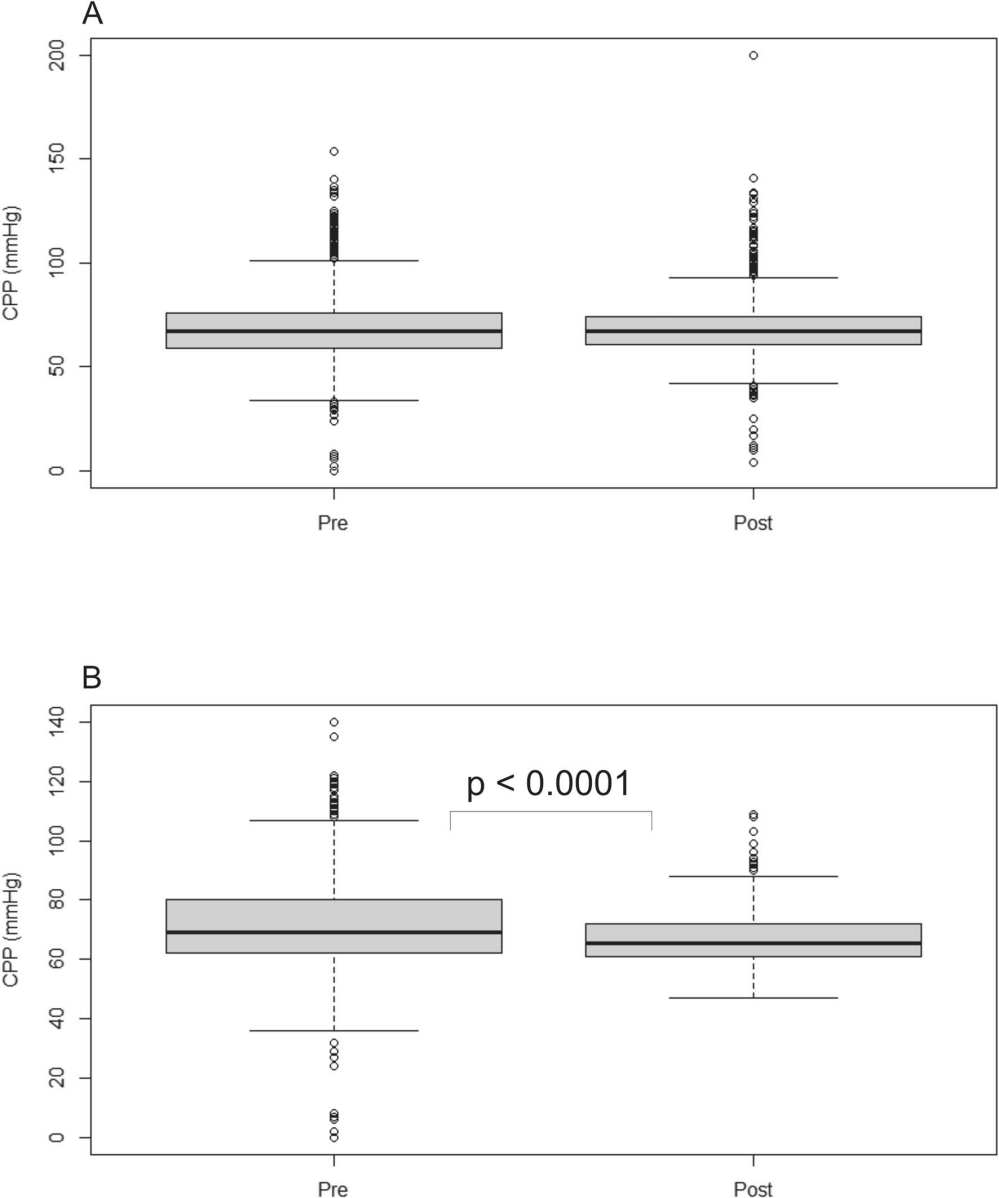
**A**, CPP values in patients treated between 2013 and 2016 (pre, 6,721 values) and 2018 and 2022 (post, 5,639 values). The median CPP was 67 mm Hg in both groups. **B**, Subgroup analysis of patients with intraventricular ICP measurement alone (21 patients in 2013–2016 and 5 patients in 2018–2022). The median CPP was 69 mm Hg before and 65.5 mm Hg after change of reference point position (*p* < 0.0001). Data are shown as median, interquartile range, and range. CPP = cerebral perfusion pressure

CPP was calculated using ICP values from intraparenchymal or intraventricular catheters, and MAP was measured by an arterial Line. One patient treated between 2013 and 2016 was excluded due to missing data. Consequently, CPP values from 48 patients between 2013 and 2016 (6,721 values) and 53 patients between 2018 and 2022 (5,639 values) were collected during the first 7 days of treatment. The median CPP was 67 mm Hg (IQR 59–76 mm Hg vs. 61–74 mm Hg) regardless of the reference point for ICP and MAP (Fig. 3). The median CPP in patients with only intraventricular ICP monitoring was 69 mm Hg (IQR 62–80 mm Hg) in patients treated between 2013 and 2016 (21 patients, 2,280 values) and 65.5 mm Hg (IQR 61–72 mm Hg) in patients treated between 2018 and 2022 (5 patients, 578 values; *p* < 0.00001).

### MAP Levels

**Fig. 4:**
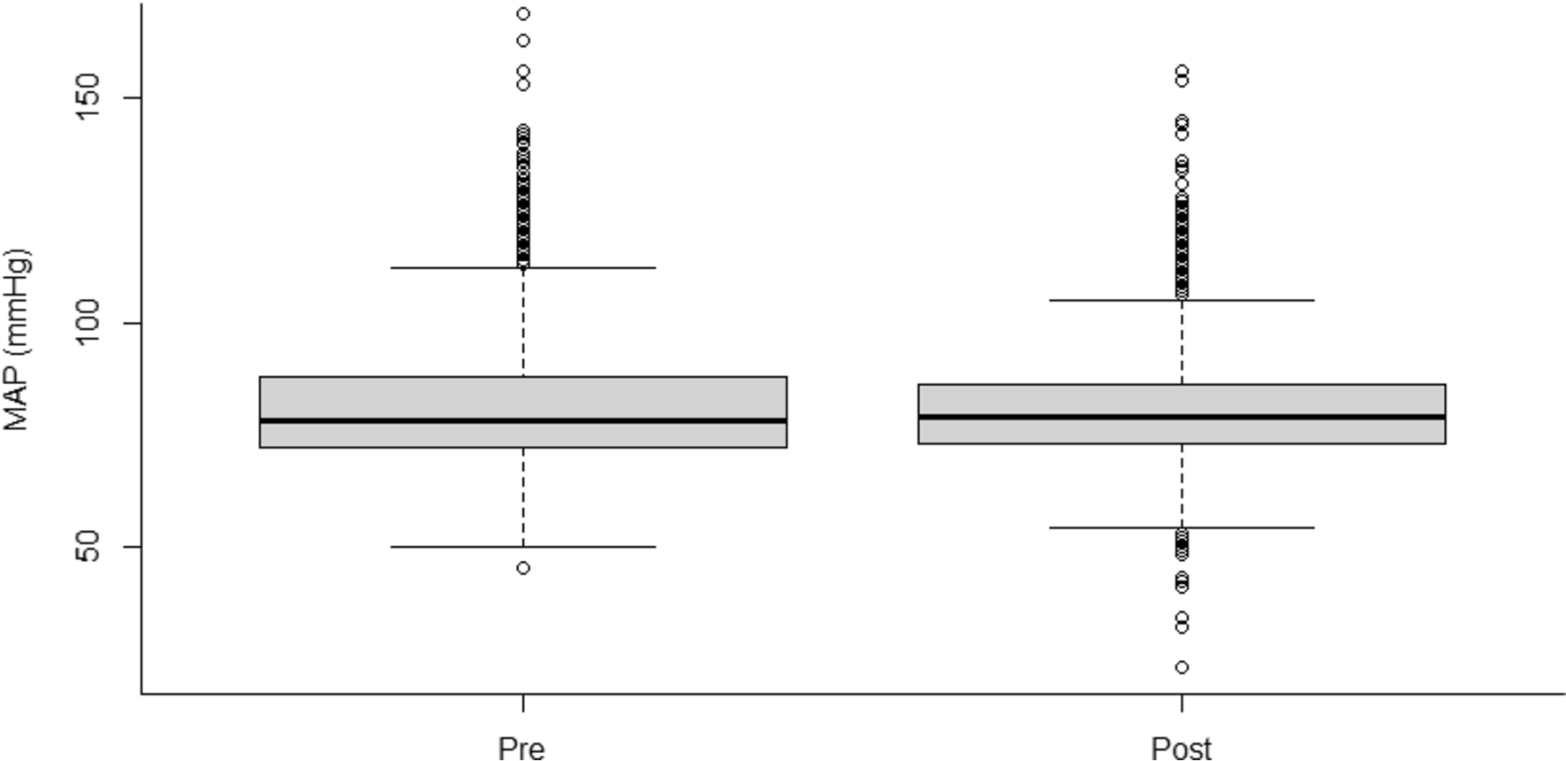
MAP values in patients treated between 2013 and 2016 (pre) and 2018 and 2022 (post). No statistically significant difference was found. Data are shown as median, interquartile range, and range. MAP = mean arterial pressure

MAP was obtained from an arterial Line in all patients during the first 7 days of treatment. We collected 7,011 MAP values from patients treated between 2013 and 2016 and 5,903 MAP values in patients treated between 2018 and 2022. No significant difference in median MAP was found between the two cohorts (Fig. 4).

### Medical Management

**Fig. 5:**
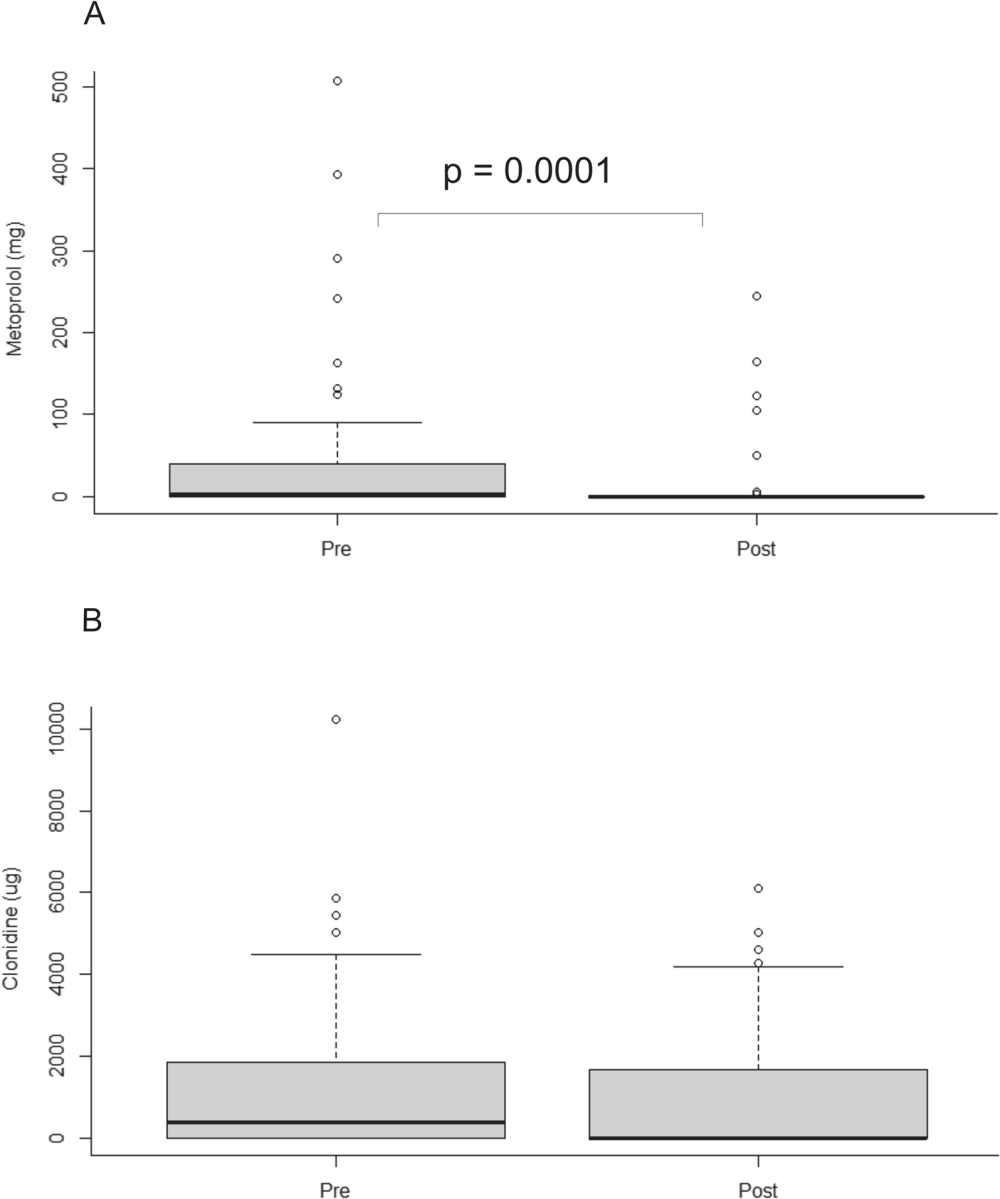
Total doses of metoprolol (**a**) and clonidine (**b**) during the entire neurocritical care unit stay in patients treated between 2013 and 2016 (**a**: 24 patients; **b**: 31 patients) and 2018 and 2022 (**a**: 7 patients; **b**: 26 patients). Although no significant differences in clonidine treatment were found, the use of metoprolol was significantly lower in patients treated after change of the arterial blood pressure transducer position in 2018–2022. Data are shown as median, interquartile range, and range

**Fig. 6:**
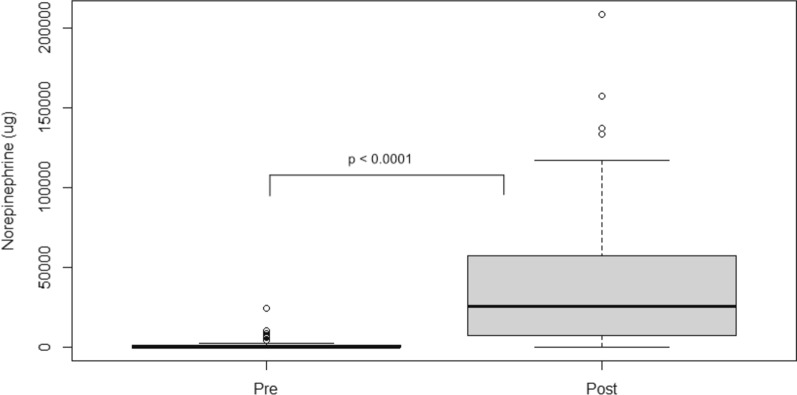
Total doses of norepinephrine during the entire neurocritical care unit stay in patients treated between 2013 and 2016 (20 patients) and 2018 and 2022 (50 patients). A statistically significant increase in total doses of norepinephrine in 2018–2022 is noted. Data are shown as median, interquartile range, and range

One patient treated between 2013 and 2016 was excluded due to missing data. In the remaining 48 patients treated between 2013 and 2016, treatment with clonidine, metoprolol, norepinephrine, and pentothal was compared to that of the 53 patients treated between 2018 and 2022. The use of barbiturates was generally infrequent, but they were used in more patients in the later cohort (2 patients in 2013–2016 vs. 9 patients in 2018–2022). There was no significant difference in either the use (65% vs. 49%; *p* = 0.17) or the doses of clonidine (median 389 µg [IQR 0–1,841 µg] vs. 0 µg [IQR 0–1,677 µg]; *p* = 0.2) (Fig. 5). However, fewer patients were treated with metoprolol (13% vs. 50%; *p* = 0.0002), and lower total doses of metoprolol (median 0 mg [IQR 0–0 mg] vs. 3 mg [IQR 0–38 mg]; *p* = 0.0001) were given to patients treated between 2018 and 2022 (Fig. 4). A marked increase was observed in both the use of norepinephrine (98% vs. 42%; *p* < 0.0001) and the total doses (median 41,723 µg [IQR 6,960–57,520 µg] vs. 0 µg [IQR 0–1,357 µg]; *p* < 0.0001) administered to patients treated between 2018 and 2022 (Fig. 6).

## Discussion

The major findings of the present study were a statistically significant increase in recorded ICP, a substantial decrease in metoprolol treatment, and a marked increase in norepinephrine use following the change of reference points for ICP and MAP from the vertex and the PA, respectively, to the EAM. The two cohorts were similar without significant differences in, for example, age, GCS, Marshall classification, or outcome. The analyses of ICP, CPP, and MAP were based on more than 31,000 values, collected every hour. The present study is the first study to comprehensively evaluate the effect of adjusting reference points for ICP and MAP/CPP on physiological values and medical management in patients with TBI.

We are not aware of any previous studies comparing ICP from different reference points in patients with TBI or any studies comparing CPP with different reference points for both ICP and MAP. However, a previous prospective study [37] demonstrated a decrease in CPP of 10–12 mm Hg when MAP was measured at the level of the tragus when compared to the PA. The study also suggested that calculating CPP from different reference points may influence clinical management decisions, length of stay, and outcome. Unfortunately, in that study, CPP was only collected once daily, and only 8.8% of patients had TBI.

The difference in median ICP was only 3 mm Hg, which is lower than anticipated. One explanation for this could be that head positioning, an important factor to consider in the assessment of ICP monitoring [25], was never accounted for. At our institution, head elevation to 30 degrees is considered standard practice and is thus commonly performed in the routine management of patients with severe TBI. However, deviations from this practice are not documented in the medical records, which represents a limitation of our study. Also, we cannot exclude the risk of inaccurate ICP calibration and/or transducer location in daily practice. Furthermore, patients treated between 2013 and 2016 also had a significantly longer NCC unit stay and longer monitoring hours. This may suggest that these patients had a higher frequency of pathological ICP values compared to those treated between 2018 and 2022, which could partially account for the relatively small difference in ICP. Although the difference in ICP was relatively small in the present study, we argue that our findings are clinically significant in this cohort of patients with severe TBI, in whom tight ICP control is crucial, as elevated ICP values can lead to detrimental physiological changes in the injured brain and may be associated with inferior outcome.

In contrast to previous studies [7, 13, 37, 38], no statistically significant difference on MAP was seen when the reference point was changed to the EAM. One potential reason for this is that medical interventions to counteract low MAP were rapidly undertaken. This statement is supported by a significant reduction in metoprolol doses, a significant increase of norepinephrine doses, and the fact that 98% of patients were treated with norepinephrine and only 13% were treated with metoprolol during their NCC unit stay after the reference point was changed to the EAM. The lack of documentation of head positioning, as previously noted, is also a limitation of our study with respect to the interpretation of MAP and, consequently, CPP. However, we did expect a difference, as standard practice involves head elevation to 30 degrees, which would, in theory, result in a significant difference in MAP when measured at the EAM compared to the PA.

We expected a decrease of approximately 10–15 mm Hg in CPP [13, 21, 23, 37] when both reference points were changed to the EAM; however, the median CPP was similar (67 mm Hg) in both cohorts. As previously stated, a change in CPP is caused by changes in ICP and/or MAP, and arguably, the reason why CPP was not decreased may be due to the subtle difference in ICP and the use of vasopressors to increase MAP, as mentioned in the previous paragraphs. In addition, CPP levels were mainly calculated from intraparenchymal sensors not affected by the change of reference points and should therefore be interpreted with caution. This statement is supported by the fact that in patients with intraventricular ICP monitoring exclusively, CPP was significantly lower when the EAM was used as the zero-reference point. However, it should be noted that in this subgroup analysis, only 5 patients from 2018–2022 were included, compared to 21 patients from 2013–2016. The difference is due to the increased use of combined ICP catheters with both intraventricular and intraparenchymal measurements during the later period.

Our present study has several limitations due to its retrospective design, including the risk of selection bias, missing data, data inaccuracy, and some patients being lost to follow-up. Unfortunately, 45% of patients received intraparenchymal sensors and could not be included in the analysis of ICP because intraparenchymal ICP values are not calibrated to the zero point of the intraventricular transducer. This explains why fewer ICP values were recorded compared to CPP and MAP values. Also, registered CPP values in the medical software were not specified as to whether they were calculated from an intraventricular catheter or an intraparenchymal sensor. However, all CPP values were matched to ICP values recorded at the same point in time. We are aware of the limitation that physiological values were only collected once every hour, and more high-frequency data could have produced different results.

We cannot exclude the possibility that changes in medical management influenced our results, as the treatment protocol was revised concurrently with the change in reference points, allowing greater use of norepinephrine overall and specifically for sepsis, a relatively common complication in patients with severe TBI [39, 40]. Historically the cornerstone of the Lund concept has been the early use of metoprolol and clonidine to reduce CPP and the avoidance of norepinephrine, primarily due to the risk of cerebral vasoconstriction [30]. Moreover, propofol, fentanyl, and midazolam—all considered standard sedative and analgesic agents in the NICU under study—are known to induce hypotension, which could, in turn, account for differences in norepinephrine use. However, because the sedation regimens were identical in both cohorts, we believe this did not significantly affect our results.

Although all these factors must be taken into consideration when interpreting the results of the present study, it is unlikely that the significant differences observed in the use of metoprolol and norepinephrine among patients treated between 2018 and 2022 can be explained solely by changes in medical management. Instead, these differences were likely attributable, at least in part, to measures taken to counteract low MAP values when the transducer was positioned at the EAM.

We concur with previous studies that emphasize the importance of reporting reference levels for ICP and MAP transducers, as it affects medical management and possibly outcome. More specifically, the markedly increased use of norepinephrine when MAP is measured at the level of the EAM, as indicated by our results, may contribute to adverse effects, such as reduced cerebral oxygenation and the development of acute respiratory distress syndrome [41, 42]. However, the most critical question remains unanswered: which strategy yields the best outcome? In the present study, the mortality rate was numerically higher (25% vs. 19%) in patients treated at the later time point (2018–2022). However, the differences in mortality and GOSE were not statistically significant, and this study was not powered for outcome and/or mortality assessment. A study investigating different reference points, adequately powered for outcome assessment, and preferably incorporating high-frequency sampling of physiological data is warranted.

## Conclusions

In this study of patients with severe TBI, higher ICP levels and lower CPP levels were observed when the reference point was changed to the EAM. Additionally, there was decreased use of metoprolol, accompanied by a substantial increase in the use of norepinephrine. These results show the importance of zero-reference point placement, which should be reported in TBI studies analyzing ICP and CPP management.
